# Metabolism of Selected 2-Arylbenzofurans in a Colon In Vitro Model System

**DOI:** 10.3390/foods10112754

**Published:** 2021-11-10

**Authors:** Ondrej Vesely, Petr Marsik, Veronika Jarosova, Ivo Doskocil, Karel Smejkal, Pavel Kloucek, Jaroslav Havlik

**Affiliations:** 1 Department of Food Science, Faculty of Agrobiology, Food and Natural Resources, Czech University of Life Sciences Prague, 16500 Prague, Czech Republic; czeveselyo@gmail.com (O.V.); jarosovav@af.czu.cz (V.J.); kloucek@af.czu.cz (P.K.); havlik@af.czu.cz (J.H.); 2 Department of Microbiology, Nutrition and Dietetics, Faculty of Agrobiology, Food and Natural Resources, Czech University of Life Sciences Prague, 16500 Prague, Czech Republic; doskocil@af.czu.cz; 3 Department of Natural Drugs, Faculty of Pharmacy, Masaryk University, 61200 Brno, Czech Republic; karel.mejkal@post.cz

**Keywords:** moracin C, mulberrofuran G, mulberrofuran Y, permeability assay, Caco-2 cells, intestinal fermentation, LC-MS-Q-TOF

## Abstract

2-arylbenzofurans represent a small group of bioactive compounds found in the plant family Moraceae. As it has not been investigated whether these substances are stable during passage through the gastrointestinal tract, their biological effects may be altered by the metabolism of intestinal microbiota or cells. The aim of the present study was to investigate and compare mulberrofuran Y (**1**), moracin C (**2**), and mulberrofuran G (**3**) in an in vitro model of human intestinal bacterial fermentation and in an epithelial model using the Caco-2 cell line. The analysis of compounds by LC-MS-Q-TOF showed sufficient stability in the fermentation model, with no bacterial metabolites detected. However, great differences in the quantity of permeation were observed in the permeability assay. Moreover, mulberrofuran Y (**1**) and moracin C (**2**) were observed to be transformed into polar metabolites by conjugation. Among the test compounds, mulberrofuran Y (**1**) was mostly stable and accumulated in endothelial cells (85.3%) compared with mulberrofuran G (**3**) and moracin C (**2**) (14% and 8.2%, respectively). Thus, only a small amount of mulberrofuran Y (**1**) was conjugated. Moracin C (**2**) and mulberrofuran G (**3**) were metabolized almost completely, with only traces of the unchanged molecule being found on the apical and cellular sides of the system. Only conjugates of mulberrofuran Y (**1**) and moracin C (**2**) were able to reach the basolateral side. Our results provide the basic description of bioavailability of these three compounds, which is a necessary characteristic for final evaluation of bio-efficacy.

## 1. Introduction

2-Arylbenzofurans are uncommon phenolic compounds that have been found only in a limited number of plant families, such as Corsiniaceae, Gnetaceae, Melanthiaceae, Stemonaceae, Moraceae, Fabaceae, and Vitaceae [1]. They are present in significant amounts in all parts of the mulberry tree (*Morus* spp.).

2-Arylbenzofurans have a structure related to stilbenoids and exhibit bioactive effects with potential applications in medicine and human nutrition [2]. The backbone of their skeleton is based on a benzofuran ring substituted with a phenyl group. Derivatives of benzofurans show potent biological activities including an antimicrobial effect [3], with some showing similar potency to commercial antibiotics [4]. They also exhibit antiparasitic [5], analgesic [6], antitumor, and kinase inhibitor activity [7]. Mulberrofuran G (syn. albanol A) showed potent antimicrobial effects [8] and acts as a COX and LOX inhibitor [9], which means that it can modulate the inflammatory response. Mulberrofuran Y (**1**) and moracin C (**2**) demonstrated cytotoxic activity using the THP-1 human monocytic leukemic cell line of 4.8 ± 0.19 and 3.2 ± 0.13 µM, respectively [10]. These compounds also significantly inhibited the production of nitric oxide in RAW 264.7 macrophage cells [11]. Other biological activities of moracin C (**2**) and mulberrofuran G (**3**), such as hypotensive, antitumor preventive, and others, have been summarized in review articles [12,13]. These results suggest a potential benefit of 2-arylbenzofurans dietary intake, especially for chronical inflammatory diseases and tumor prevention [9,13].

In plants, phenolic compounds are commonly present as part of higher molecular structures. They form polymeric structures with other phenolics, which are bound to other organic substances including dietary fibers, proteins, saccharides, and organic acids. This could lead to a relatively low absorption rate of plant phenolic compounds in the human small intestine, with approximately 5–10% of all of them being digested in the proximal part of the gastrointestinal tract, and the remainder passing to the colon where they can be fermented by bacteria [14,15]. Once in the colon, polyphenols are metabolized by the gastrointestinal microbiota to compounds of lower molecular weight, such as phenolic acids, which can be readily absorbed across the intestinal barrier into the bloodstream. In general, for phenolic compounds, there are three well-documented major pathways for most plant metabolites: hydrolysis, cleavage, and reduction [16].

Although information on the metabolism of a number of important polyphenolic compounds by the human gut microbiota is becoming increasingly available, much is still unknown for 2-arylbenzofurans. For this reason, we attempted to investigate the fate of selected 2-arylbenzofurans in the gut based on the hypothesis that these compounds are degraded in the colon by the gut microbiota in a manner similar to that of other structurally or biochemically related phenolic compounds. Because the bioactive compounds including 2-arylbenzofurans can be transformed into metabolites with changed activity or may be retained inside epithelial cells during their transport across the intestinal barrier, it is important to investigate their fate in an in vitro permeability model. This may also indicate the potential site of action of ingested 2-arylbenzofurans. To this end, we used two methods: an in vitro fermentation model to screen for degradation of mulberrofuran Y (**1**), moracin C (**2**), and mulberrofuran G (**3**), and a permeability assay on the Caco-2 cell line in Transwell^TM^ plates to screen for the intestinal metabolism of these compounds.

## 2. Materials and Methods

### 2.1. Study Design

Using a batch fermentation model of the human colon, 2-arylbenzofurans were incubated after the addition of human fecal bacteria from three donors. Samples were collected at 0, 2, 4, and 8 h and the effect of microbiota on tested 2-arylbenzofurans was determined by UHPLC/MS HRAM.

### 2.2. Ethics Statement and Donor Information

All three volunteers (two males and one female aged 23, 28, and 29 years) were asked to donate stool samples free of charge. The ethical agreement for stool collection was obtained by the ethical committee (ZEK/22/09/2017) of the Czech University of Life Sciences in Prague. Body mass index was estimated to be between 23 and 26.5. Because we wanted to limit dietary interference, the donors were forced to follow a low-polyphenol diet for at least 2 days before starting the experiment. No antibiotics were allowed for at least 3 months before the sample collection. They had no history of chronic gastrointestinal disease. The woman was neither breastfeeding nor pregnant. Samples were collected from November to December and analyzed at the Czech University of Life Sciences Prague.

### 2.3. Reagents

**Figure 1:**
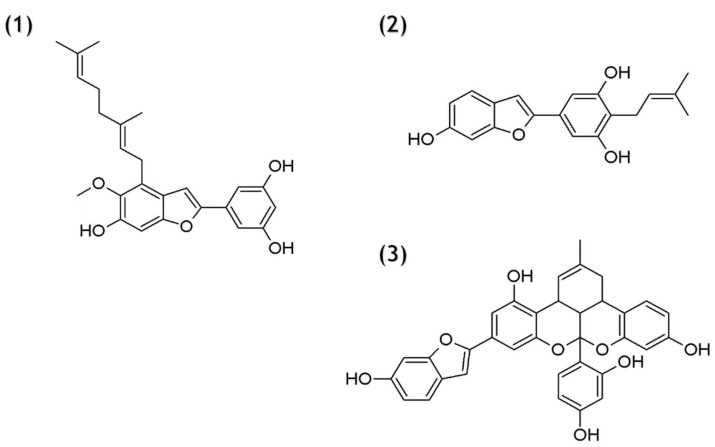
Structure of the tested compounds mulberrofuran Y (**1**), moracin C (**2**), and mulberrofuran G (**3**).

The fermentation medium was prepared from chemicals obtained from Merck (Darmstadt, Germany). Standards: 2-arylbenzofurans mulberrofuran Y (**1**), moracin C (**2**), and mulberrofuran G (**3**) were obtained from ChemFaces (WuHan, China) in 98% purity (Figure 1). The organic solution, ethyl acetate, and methanol were purchased from the VWR International (Stribrna Skalice, Czech Republic) in the analytical grade. Dimethylsulphoxide was obtained from VWR Chemicals. Formic acid was obtained from Fisher Scientific (Merelbeke, Belgium) in >98% purity. Ultra-pure water was obtained from a Millipore system (Bedford, MA, USA).

### 2.4. Fermentation Medium

Medium was prepared from 225 mL of distilled water, 112.5 mL of macromineral solution, 56.25 μL of micromineral solution, 562.5 μL of 0.1% resazurin solution, and 1.125 g of tryptone. The macromineral solution was prepared from 7.14 g of Na_2_HPO_4_, 6.2 g of KH_2_PO_4_, 0.6 g of MgSO_4_, and up to 1 L of distilled water. The micromineral solution was made of 2.64 g of CaCl_2_, 2 g of MnCl_2_, CoCl_2_, 1.6 g of FeCl_3_, and up to 20 mL of distilled water. CO_3_ buffer was created by mixing 1 g of NH_4_HCO_3_, 8.75 g of NaHCO_3_, and distilled water up to 250 mL.

### 2.5. Phosphate Buffer and Reducing Solution

Phosphate buffer for fecal slurries was created by dissolving 1.77 g of KH_2_PO_4_ in 195 mL of distilled water and 3.62 g of Na_2_HPO_4_ in 305 mL of distilled water (both 1/15M). The acidity of the mixed phosphate buffer was adjusted to a neutral pH by hydrochloric acid. The reducing solution was created by mixing 125 mg of cysteine hydrochloride, 125 mg of Na_2_S, 0.8 mL of 1M NaOH, and distilled water up to 20 mL.

### 2.6. Fermentation with Human Fecal Microbiota

Mulberrofuran Y (**1**), moracin C (**2**), and mulberrofuran G (**3**) were dissolved in DMSO to a final concentration of 10 mg/mL. After boiling the fermentation medium and sodium phosphate buffer, both were cooled to 37 °C. To remove oxygen, the samples were purged with nitrogen gas (approximately 30 min). The pH of the medium was equilibrated to neutral pH using HCl. For each fermentation bottle, 17.18 mL of fermentation medium and 0.8 mL of reducing solution were transferred to create fermentation vials. Fecal slurry was prepared immediately after sampling from each donor by homogenizing the collected feces in a stomacher bag with phosphate buffer to produce a 25% fecal slurry. After filtration through the mesh, 2 mL of the filtered fecal slurry were added to each fermentation bottle. Finally, 20 μL of the tested compound or DMSO as a negative control were added. The bottles were incubated at 37 °C for 8 h with stirring at 100 strokes per minute. Aliquots of fecal suspensions were collected at 0, 2, 4, and 8 h, and stored at −80 °C until further analysis.

### 2.7. Samples Purification

First, 400 μL sample of supernatant fluid were defrosted and centrifuged (5 min/15,000 RPM) and the pellet was erased. Supernatant was combined with 20 μL of ^13^C_6_ *trans*-resveratrol in methanol (2 μg/mL), which was used as an internal standard. Samples were extracted 3 times with 2.5 mL of ethyl acetate and 2 mL of ultra-pure water. After liquid-liquid extraction, ethyl acetate was evaporated with nitrogen gas and redissolved in 1 mL of methanol (VWR Chemicals, Stribrna Skalice, Czech Republic) in combination with formic acid (Fisher Scientific, Merelbeke, Belgium) (1%).

### 2.8. Metabolite Analysis

The analysis was performed on an LC/MS system consisting of an Ultimate 3000 UPLC chromatograph Thermo Fisher Scientific (Waltham, MA, USA) coupled to an Impact II ultra-high resolution high mass accuracy mass spectrometer (HRAM) Q-TOF (Bruker Daltonics, Bremen, Germany) equipped with an electrospray ionization (ESI) source.

The separation was carried out on a Phenomenex F5 Kinetex column (1.7 μm 100 Å 100 × 2.1 mm) (Phenomenex, Torrance, CA, USA) by using a mobile phase consisting of 0.1% formic acid (solvent A) and methanol (solvent B). The binary gradient was run at a flow rate of 0.2 mL/min as follows: 0–3 min isocratic at 20% B, 3–6 min from 20% to 50% B, 6–15 min isocratic at 100% B, and 15–20 min isocratic at 20% B. The column oven was adjusted to 35 °C, and the injection volume was 5 μL.

For metabolite screening, the ESI source was set in both positive and negative mode. Semiquantitative analysis was performed in negative mode with the parameters listed in the Appendix A. Each compound was confirmed by MS/MS fragmentation with a collision energy voltage (20, 30, and 50 eV). Data acquisition was performed using HyStar 3.2 SR4, Otof series 4.0 (Bruker Daltonics, Bremen, Germany) and Chromeleon Xpress (Thermo Fisher Scientific, Waltham, MA, USA) software and the data obtained were analyzed by using DataAnalysis 4.3. (Bruker Daltonics, Bremen, Germany). Commercially available standards of mulberrofuran Y (**1**), moracin C (**2**), and mulberrofuran G (**3**), each at 9 concentration levels ranging from 0.5–500 ng/mL, were used for calibration. A list of the 2-arylbenzofurans monitored in the samples is given in the Appendix B.

### 2.9. Permeability Experiment

The Caco-2 cell lines were purchased from the American Type Tissue Collection (Rockville, MD, USA). Caco-2 cells in Transwells at passage 25 were used. More information on cell culture and passaging is described in [17].

### 2.10. Preparation of Inserts

Cells were cultured in DMEM-F12 (Dulbecco’s modified Eagles medium—Nutrient Mixture F-12) supplemented with 10% FBS (fetal bovine serum), 1% nonessential amino acids, 1% penicillin, and streptomycin, all obtained from Sigma-Aldrich (Prague, Czech Republic) in humidified air containing 5% CO_2_. Cells were seeded at a density of 2.6 × 10^5^ cells/cm in 24-wells. The inserts were pre-filled with 50 µL of medium before seeding the cells. After seeding, the basolateral side of the chamber was filled with 1 mL of DMEM, and cells were grown in a humidified atmosphere of 5% CO_2_ at 37 °C. Non-adherent cells were removed by removing the medium after 6 h of incubation and replaced with 0.5 mL of fresh DMEM. The culture medium was replaced seven times a week for 21 to 25 days prior to the transport experiment.

### 2.11. Measuring of the Monolayer Integrity

The filter inserts containing a monolayer of the Caco-2 cell line were washed three times with HBBS (Hanks’ Balanced Salt solution) heated to 37 °C and pH 7.4. The integrity of the cell monolayer was confirmed by measuring the transepithelial electrical resistance (TEER), which had to be at least 600 Ω. After adding 25 µM lucifer yellow, the plates were incubated at 37 °C in 5% CO_2_ atmosphere for 1 h with shaking (150 rpm). Using a Tecan Infinite M200 reader (Excitation/Emission wavelength 480 nm/530 nm), the plates were measured. Only plates with an integrity greater than 95% were used in the following experiment.

### 2.12. Metabolism and Absorption of 2-Arylbenzofurans

Initially, 500 µL of 20 µM test substance solutions were added to the apical side of each insert and 1000 µL of HBSS were added to the basolateral side. At the same time, 50 µL were taken immediately from the apical side at the time point of 0 h. For future analysis, basolateral sampling times of 0.5, 1, 1.5, 2, 3, and 4 h were chosen while placing the plates in the incubator and using an orbital shaker (150 rpm). In this experiment, 500 µL of sample were always taken from the basolateral side and replaced with an equal amount of pure HBSS. After 4 h, the apical side was simultaneously removed, and the inserts were washed three times with HBSS. The TEER method was again used to verify that the experiment did not compromise cellular integrity. Finally, cell integrity was disrupted by adding 100% methanol and the cell contents with the other samples were stored at −80 °C until the analysis.

### 2.13. Statistical Evaluation

Due to the design of the experiment and the decreasing concentration of substances on the basolateral side, it was necessary to recalculate each time point by the following equation:

$$C_{A}=\frac{\sum^{\;}C_{P}}{2}+C_{M}$$

where *C_A_* is the actual concentration at the time point, *C_P_* are the previous concentrations, and *C_M_* is the concentration measured at the time point. Three biological repetitions were performed for all compounds. Samples were measured by LC/MS in triplicates. Results are presented as a mean ± standard error. Quantitative data were normalized to 20 µM to correct the minor dilution errors. Graph creation and basic statistical analysis were carried out via Microsoft Excel and SPSS version 25 (IBM Corp., Armonk, NY, USA).

## 3. Results

### 3.1. Fermentation Study

Mulberrofuran Y (**1**), moracin C (**2**), and mulberrofuran G (**3**) were tested for their metabolic behavior in the colonic microbial environment in vitro. The identification and comparison of the samples with the standards is given in the Appendix B.

The intestinal bacterial fermentation in vitro assay was performed using feces from three donors (D) as inoculum for the analysis of the metabolism of selected compounds, (mulberrofuran Y (**1**), moracin C (**2**), mulberrofuran G (**3**)). As shown in Figure S1, significant differences in microbial transformation were observed between 2-arylbenzofurans. No metabolites were detected. The amount of parent compounds was monitored: after 8 h, the concentration of moracin C (**2**) decreased from an initial 10 μg/mL to 7 to 9 μg/mL depending on the donor. In contrast, mulberrofuran G (**3**) was degraded by half to 5 μg/mL in 2 donors and was stable in one donor. In this particular case, the minimum was detected at 2 h (9 μg/mL), was stable at 4 h, and reached 10 μg/mL again at 8 h. Mulberrofuran Y (**1**) was continuously degraded to between 2.5 and 6.9 μg/mL depending on the donor.

Minor inter-individual differences were observed among the donors in the moracin C group, compared to mulberrofuran G and mulberrofuran Y, as Figure S1 shows.

### 3.2. Permeability Study

**Figure 2:**
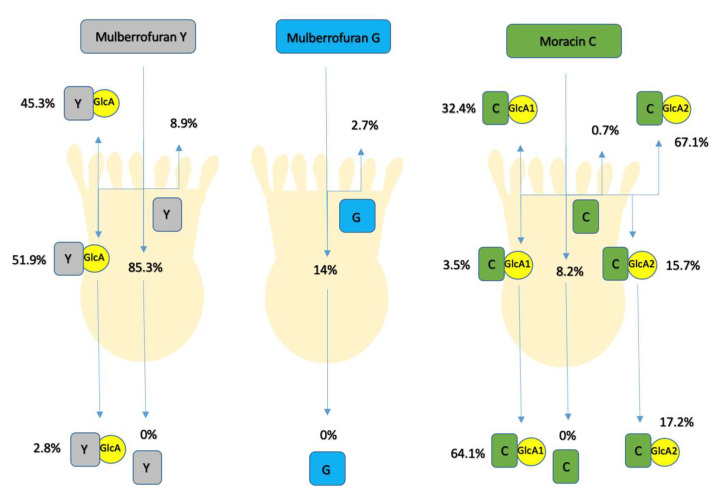
The fate of mulberrofuran Y (**1**), moracin C (**2**), and mulberrofuran G (**3**) after 4 h of the experiment in the Transwell^TM^ cell system. Y: Mulberrofuran Y (**1**), G: Mulberrofuran G (**3**), C: Moracin C (**2**), GlcA: Glucuronide conjugation.

The transcellular transport of compounds in Caco-2 cells was investigated using an in vitro model of the human intestinal barrier. The overall and simplified scheme is described in Figure 2.

Samples were measured on the LC/MS system as it was described previously. Table 1 lists the four main pathways of the monitored compounds. Of the initial 20 μM as 100%, only mulberrofuran Y (**1**) was mostly stable (94. 2 ± 25.8%) and accumulated in intestinal cells (85.3 ± 19.9%). A small volume (8.9 ± 5.9%) was detected on the apical side that was unchanged. The remainder (5.8 ± 25.8%) was converted or metabolized. In contrast, mulberrofuran G (**3**) and moracin C (**2**) were predominantly transformed or metabolized by the cells (83.3 ± 1.1% and 91.1 ± 0.7%, respectively). Only traces were detected on the apical side (2.7 ± 0.5% and 0.7 ± 0.2%, respectively) and cells (14.0 ± 0.6% and 8.2 ± 0.5%, respectively) after 4 h of incubation. Interestingly, none of the parent compounds were found on the basolateral side in the experiment.

**Figure 3:**
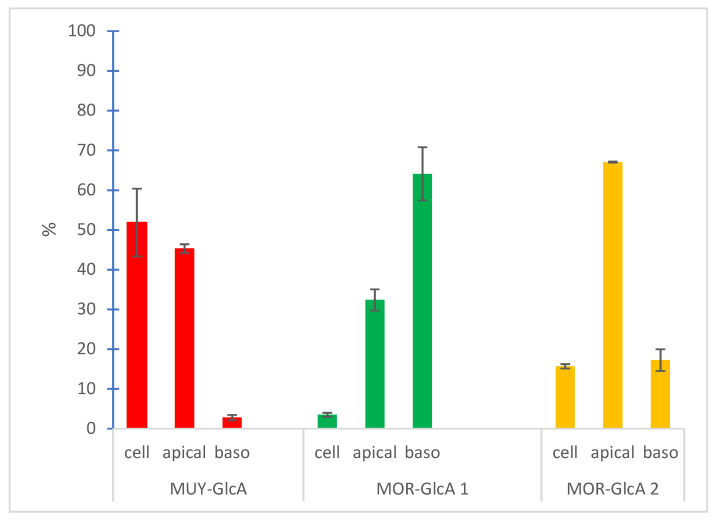
Distribution of main glucuronide conjugates in the in vitro permeability model (Transwell^TM^ Caco-2 cell system) among cell, apical, and basolateral compartments observed after 4 h of incubation. Values obtained from LC/MS for compounds are expressed as ratios of compounds to internal standard (ISTD) as means ± 1 SE (*p* > 0.05), standard deviation, *n* = 3. MUY-GlcA: mulberrofuran Y—GlcA, MOR-GlcA 1: moracin C—GlcA 1, MOR-GlcA 2: moracin C—GlcA 2.

Similarly, only the glucuronide conjugates of mulberrofuran Y (**1**) and moracin C (**2**) were found, as shown in Figure 3. The glucuronide of mulberrofuran Y (**1**) (RED) became detectable on the basolateral side after 3 h of incubation. In summary, after 4 h of incubation, the main volume of mulberrofuran Y glucuronide was found inside cells (51.9 ± 8.5%) and the remainder on the apical and basolateral sides (45.3 ± 1.1% and 2.8 ± 0.7%, respectively). Moracin C was also metabolized only by conjugation with glucuronic acid as mulberrofuran Y, but unlike the previous one, two different conjugates were formed. The first of these (MOR-GlcA 1) was detected on the basolateral side in the first 30 min, while the second (MOR-GlcA 2) became detectable after 1 h of incubation. Both metabolites were detectable inside the cells (3.5 ± 0.5% and 15.7 ± 0.6%, respectively) and on the apical side (32.4 ± 2.7% and 67.1 ± 0.2%, respectively) after 4 h of incubation. Steric positions of bound conjugated units on MUG-GlcA, MUY-GlcA, MOR-GlcA 1, and MOR-GlcA 2 cannot be specified due to the lack of confirmed standards. Mulberrofuran G (**3**) was hardly metabolized in this model; only traces of its glucuronide conjugate were detected in the cell fraction after 4 h of incubation.

The glucuronide of mulberrofuran Y was detected in all monitored fractions. Most of it was found inside the cells and on the apical side. Both glucuronide conjugates of moracin C were detected mainly on the apical side. In addition, there are differences between the ratios on the basolateral side and in the cells. The first metabolite of moracin C (MOR-GlcA 1) tended to occur on the basolateral side, and the second (MOR-GlcA 2) preferentially on the apical side, as Figure 3 shows.

All basolateral metabolites are shown in a cumulative plot (Figure S2). The basolateral concentration of mulberrofuran Y (**1**) started after 3 h and increased continuously throughout the experiment.

## 4. Discussion

In the literature, substances like 2-arylbenzofurans are reported to have promising bioactive effects with potential for applications in medicine and human nutrition. This study attempted to add to the unknown information on the biotransformation of mulberrofuran Y (**1**), moracin C (**2**), and mulberrofuran G (**3**) by human microbiota and cellular uptake through an intestinal in vitro model. Bacteria did not produce any metabolites of the three compounds investigated. Moreover, the results clearly indicate a difference in metabolite sulfation and glucuronidation processes when investigating the permeability by intestinal cells.

To find out how and whether these substances can be metabolized by bacterial communities in the human gut, the tested 2-arylbenzofurans were fermented by human fecal microbiota. No metabolites were found. It has been reported in the literature that benzofuran can be cleaved at the oxygen bond in its heterocycle [18,19]. Oberoi et al. described the aerobic bacterial decomposition of benzofuran to carbon dioxide and water, with either catechol or salicylic acid as possible intermediates [18]. These products have also been observed after metabolism in flavonol compounds [20]. Similar results were described for heterocyclic O-bound cleavage of dibenzofuran when 2-hydroxybenzoic and 2,5-dihydroxybenzoic acid were formed as the major products on the O-heterocycle [19]. In this study, no common microbiota degradation products, such as 4-hydroxybenzoic acid, 3,5-dihydroxybenzoic acid, protocatechuic acid, resorcinol, or phloroglucinol, were found. This suggests that the metabolism of these compounds occurs in an unusual way or not at all. There is an assumption that the decreasing concentrations are due to the intestinal microbiota, so differences among donors could be caused by differences in the microbiota composition [21]. In our study, 2-arylbenzofurans were gradually catabolized, and their final concentration, after 8 h of fermentation, ranged from 87 ± 10% (D2) to 66 ± 3% (D1) for moracin C (**2**), 69 ± 8% (D2) to 25 ± 0% (D1) for mulberrofuran Y (**1**), and 102 ± 13% (D2) to 48 ± 0% (D1). These results clearly indicate that microbiota composition is an important aspect affecting the stability of 2-arylbenzofurans.

In contrast to our findings in bacterial exposure, human colonocytes have the ability to transform 2-arylbenzofurans. Metabolism by enterocytes is an important step affecting their bioactivity. We observed major differences among each of these compounds.

The main amount of mulberrofuran Y (**1**) (85.3 ± 19.9%) was detected to be unchanged in the cells after 4 h of incubation. This indicates that the intestinal wall is the main target site of this compound. After 4 h of incubation, 8.9 ± 5.9% of unchanged mulberrofuran Y (**1**) was found in the medium in the apical chamber. Interestingly, no amount of this compound was found on the basolateral side. However, its glucuronic conjugate was detected predominantly inside the cells and on the apical side, and only a small amount of this metabolite was detected on the basolateral side. Intestinal cells have an enzymatic system that allows them to produce two types of metabolites. Metabolites formed by conjugation with glucuronic acid or with a sulfonic acid functional group are produced by the action of the enzymes UDP-glucuronosyltransferases (UGTs) and sulfotransferases (SULTs) [22,23]. UGTs are the primary phase II enzymes catalyzing the conjugation of glucuronic acid to xenobiotics with polar groups to facilitate their clearance [23]. Our data suggest that UGTs are probably the main enzyme system included in the metabolism of mulberrofuran Y. In addition, the metabolites probably leave the cells via ABC transporters, such as multidrug resistance protein (MRP2-apical side, MRP3-basolateral side) and breast cancer resistance protein (BRCP), similar to other phenolics [24,25].

The metabolism of mulberrofuran G (**3**) differed significantly from mulberrofuran Y (**1**). Only 14.0 ± 0.6% of the parental compound was found in the cells, and 2.7 ± 0.5% on the apical side at the end of the experiment. This means that the majority of compound (83.3 ± 1.1%) was changed and nothing of the original molecule was able to reach the basolateral side. Only a small volume of glucuronic metabolite was detectable in the cells after 4 h of incubation. These results point to a significant lowering of the original molecule’s bioavailability. However, since we did not find all the metabolites, we cannot decipher the main site of action for these substances.

After application of moracin C (**2**) on Caco-2 cells, the vast majority, 91.1 ± 0.7%, were transformed. Interestingly, two types of glucuronic metabolites were detected in the cells, on the apical and basolateral sides. While in the case of mulberrofuran Y (**1**), we were able to find the metabolite mostly in the cellular region, in the case of moracin C (**2**), both metabolites favored the apical side, and then the basolateral over the cellular region. The two detected types of glucuronic metabolites can be explained by the composition of UGT (UGT1A1, 1A4, 2B7, 2B15, and others are abundant in the gastrointestinal tract) [23]. These metabolites also differ from each other in the use of ABC transporters. The result shows that the first metabolite passed to the apical side at a lower percentage (32.4 ± 2.7%) than to the basolateral side (64.1 ± 6.7%). The second metabolite was accumulated preferentially at the apical side (67.1 ± 0.2%), followed by the basolateral side (17.3 ± 2.7%). This could be due to a preference for the BRCP or MRP2. Finally, tracking the timeline of metabolite passage to the basolateral side shows that in the first 30 min of the experiment, the first metabolite can be detected. In contrast, the second metabolite began to be released after 1 h.

## 5. Conclusions

In conclusion, we explored the intestinal initial-passage metabolism of mulberrofuran Y (**1**), moracin C (**2**), and mulberrofuran G (**3**). Our experiment found significant differences in the metabolism of 2-arylbenzofurans. Our findings suggest that intestinal epithelial cells could be the main target of mulberrofuran Y (**1**) based on the Transwell^TM^ system using the Caco-2 cell line, while the intestinal microbiota represent an important factor in reducing the content of mulberrofuran G (**3**) and moracin C in the gut. The compounds differed in the degree of their conversion in the intestinal model: mulberrofuran Y (**1**) was mostly stable, and mulberrofuran G (**3**) and moracin C (**2**) were almost completely metabolized. Furthermore, we detected four metabolites. Only two glucuronic conjugates of moracin C (**2**) and one of mulberrofuran Y (**1**) were detected at the apical, basolateral, and intracellular fractions. These results provide important information on the initial bioavailability of 2-arylbenzofurans, which significantly influences their resulting bio-efficacy.

## Figures and Tables

**Table 1:** List of 2-arylbenzofurans monitored and detected in the Transwell^TM^ cell system by the LC/MS system. Mulberrofuran G (**3**) and moracin C (**2**) were mostly metabolized. Mulberrofuran Y (**1**) was stable and most of it was retained in the cells. All compounds were able to pass into cells but not to the basolateral side in their original form.

	Mulberrofuran Y (1)	Moracin C (2)	Mulberrofuran G (3)
Apical side	8.9 ± 5.9%	0.7 ± 0.2%	2.7 ± 0.5%
Basolateral side	0 ± 0%	0 ± 0%	0 ± 0%
Cells	85.3 ± 19.9%	8.2 ± 0.5%	14.0 ± 0.6%
Transformed	5.8 ± 25.8%	91.1 ± 0.7%	83.3 ± 1.1%

## Data Availability

Data is contained within the article.

## Supplementary Materials

The following are available online at https://www.mdpi.com/article/10.3390/foods10112754/s1, Figure S1: Conversion of 2-arylbenzofurans during in vitro batch fermentation with human fecal suspensions, Figure S2: Monitored trends in metabolites on the basolateral side of inserts in the TranswellTM cell system.

## Appendix A

**Table A1:** Limit of detection (LOD) was calculated as signal to noise ratio 3:1, limit of quantification (LOQ) was calculated as signal to noise ratio 10:1. N/A = not available. For the apical side of the permeability assay, the LOD and LOQ were 10-fold higher due to aliquot sampling.

Experiment	Compound	Calibration Curve Equation	R^2^	Linear Range [ng/mL]	LOD [ng/mL]	LOQ [ng/mL]	RSD [%] of Injection Triplicate
Fermentation	MUG	*Y* = 9327.3*X* + 89476	0.9951	1–500	9.70	9.94	0.54
	MUY	*Y* = 16081*X* − 585507	0.9891	50–500	36.47	36.61	N/A
	MOR	*Y* = 13991*X* + 134182	0.9951	1–500	9.66	9.82	N/A
Permeability	MUG	*Y* = 5770.9*X* + 15367	0.9947	1–500	2.83	3.22	5.07
	MUY	*Y* = 8703.5*X* + 45177	0.9941	1–500	5.30	5.55	1.07
	MOR	N/A	N/A	N/A	N/A	N/A	2.72

## Appendix B

**Table A2:** List of the compounds monitored and detected in the samples by the LC/MS system.

Compound	Retention Time (min)	Neutral Molecule Exact Mass	Measured [M-H]- Exact Mass	Comparison with Standard
Moracin C (**2**)	11.1	310.12	309.11	yes
Mulberrofuran G (**3**)	11.3	562.16	561.15	yes
Mulberrofuran Y (**1**)	12.9	408.19	407.19	yes
Moracin C-GlcA 1	10.1	486.15	485.145	no
Moracin C-GlcA 2	10.7	486.15	485.145	no
Mulberrofuran G-GlcA	11.0	738.19	737.187	no
Mulberrofuran Y-GlcA	12.2	584.23	583.218	no
