# Supplementary material for: Metabolism of Selected 2-Arylbenzofurans in a Colon In Vitro Model System
Source: Foods. 2021 Nov 10;10(11):2754. doi: 10.3390/foods10112754 (PMC8625357; doi:10.3390/foods10112754)
Supplement: Supplementary file 1 [file foods-10-02754-s001.zip › foods-1424795-SI.pdf]

Supplementary Materials

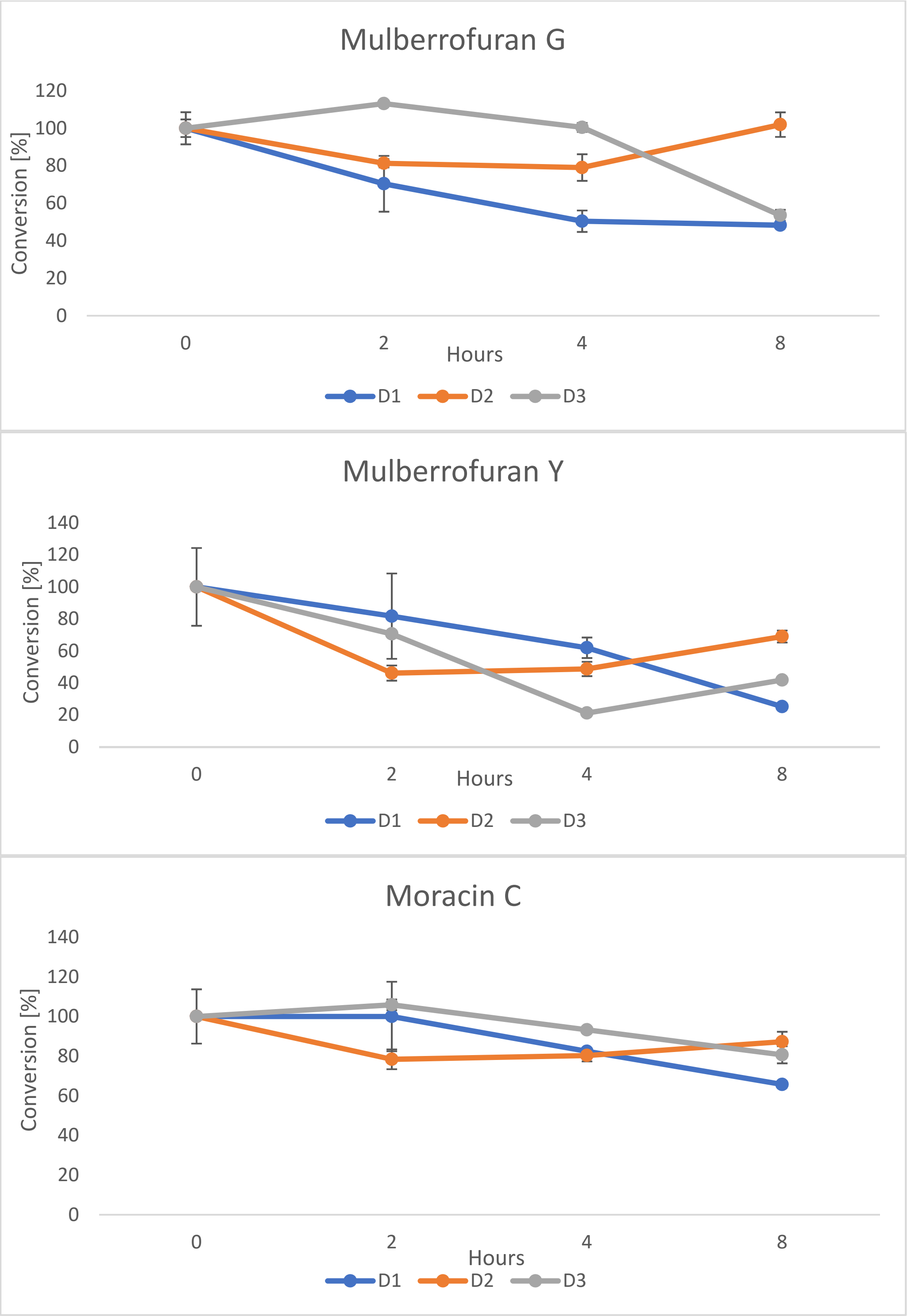

Figure S1. Conversion of 2-arylbenzofurans during *in vitro* batch fermentation with human fecal suspensions. Human fecal cultures consisted of 10% human fecal suspensions in medium containing 10 µg/mL of the compound and incubated for 8 h at 37 °C under anaerobic conditions. Values shown are arithmetic means (±SDs), conducted in triplicate. Curves are given for each subject. A: degradation of mulberrofuran G, B: decomposition of mulberrofuran Y, C: degradation of moracin C.

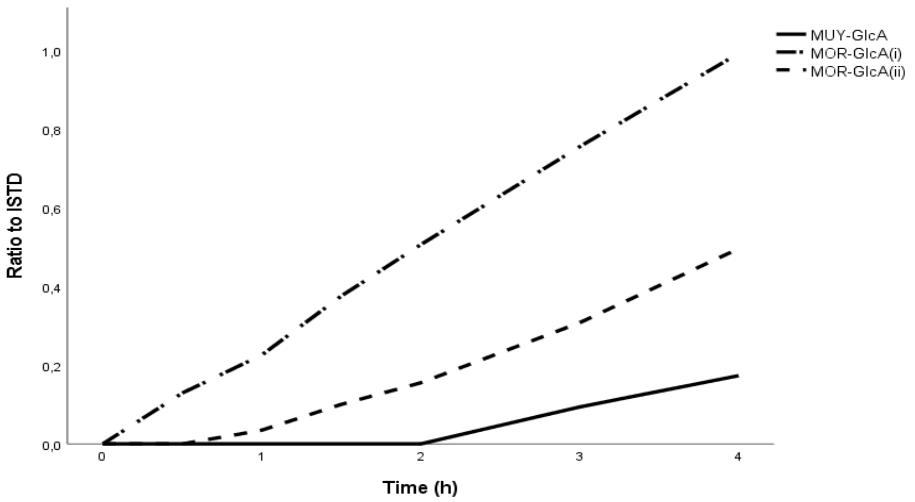

Figure S2. Monitored trends in metabolites on the basolateral side of inserts in the Transwell™ cell system. Values obtained from LC/MS for MUY-GlcA, MOR-GlcA(i) and MOR-GlcA(ii) are expressed as mean intensity ± standard deviation, n = 3.
